# *Shigella sonnei* utilises colicins during inter-bacterial competition

**DOI:** 10.1099/mic.0.001434

**Published:** 2024-02-20

**Authors:** P. B. Leung, X. M. Matanza, B. Roche, K. P. Ha, H. C. Cheung, S. Appleyard, T. Collins, O. Flanagan, B. S. Marteyn, A. Clements

**Affiliations:** ^1^​ Department of Life Sciences, South Kensington Campus, Imperial College London, London, SW72AZ, UK; ^2^​ Universite de Strasbourg, Institut de Biologie Moléculaire et Cellulaire, CNRS UPR9002, F-67000 Strasbourg, France; ^3^​ University of Strasbourg Institute for Advanced Study (USIAS), F-67000 Strasbourg, France; ^4^​ Institut Pasteur, Université de Paris, Inserm U1225, Unité de Pathogenèse des Infections Vasculaires, F-75015 Paris, France

**Keywords:** anti-bacterial activity, colicins, Interbacterial competition, polymicrobial, Shigella, T6SS

## Abstract

The mammalian colon is one of the most densely populated habitats currently recognised, with 10^11^–10^13^ commensal bacteria per gram of colonic contents. Enteric pathogens must compete with the resident intestinal microbiota to cause infection. Among these enteric pathogens are *Shigella* species which cause approximately 125 million infections annually, of which over 90 % are caused by *Shigella flexneri* and *Shigella sonnei. Shigella sonnei* was previously reported to use a Type VI Secretion System (T6SS) to outcompete *E. coli* and *S. flexneri* in *in vitro* and *in vivo* experiments. *S. sonnei* strains have also been reported to harbour colicinogenic plasmids, which are an alternative anti-bacterial mechanism that could provide a competitive advantage against the intestinal microbiota. We sought to determine the contribution of both T6SS and colicins to the anti-bacterial killing activity of *S. sonnei*. We reveal that whilst the T6SS operon is present in *S. sonnei,* there is evidence of functional degradation of the system through SNPs, indels and IS within key components of the system. We created strains with synthetically inducible T6SS operons but were still unable to demonstrate anti-bacterial activity of the T6SS. We demonstrate that the anti-bacterial activity observed in our *in vitro* assays was due to colicin activity. We show that *S. sonnei* no longer displayed anti-bacterial activity against bacteria that were resistant to colicins, and removal of the colicin plasmid from *S. sonnei* abrogated anti-bacterial activity of *S. sonnei*. We propose that the anti-bacterial activity demonstrated by colicins may be sufficient for niche competition by *S. sonnei* within the gastrointestinal environment.

## Introduction

*Shigella* is a major causative agent of bacterial dysentery (shigellosis). It remains a major cause of infant morbidity and mortality for children under the age of five, especially in resource-limited nations [1]. In resource-rich nations, *Shigella* has become an emerging pathogen of concern due to extensive and expanding resistance to multiple classes of antimicrobial drugs [2–9]. *Shigella* is spread via the faecal-oral route, most commonly through contaminated food and water sources but also via fomites. *Shigella* is thought to be a successful human-adapted pathogen due to its low infectious dose required to start an infection: this is estimated to be between 10 and 500 colony-forming units (CFUs) [10]. *Shigella* is not known to have animal or environmental reservoirs unlike many other human enteric pathogens.

The genus *Shigella* is composed of four species: *S. boydii, S. dysenteriae, S. flexneri* and *S. sonnei*. All four species have their own characteristics and epidemiology [11, 12]. The majority of contemporary cases are caused by *S. flexneri* and *S. sonnei*. These two species account for over 90 % of all shigellosis cases [13]. However, an interesting pattern has emerged where the infection ratio of these two species is inversed in middle-high income countries (low *S. flexneri*: high *S. sonnei*) when compared to low-income countries (high *S. flexneri*: low *S. sonnei*). Therefore, as a country undergoes economic improvements, *S. sonnei* displaces *S. flexneri* as the primary causative agent of shigellosis [1, 8, 14, 15]. Several theories have emerged as to why this shift occurs, with one theory focusing on how *S. sonnei* interacts with competitors within a polymicrobial environment.

Whilst there are a number of differences between *S. sonnei* and *S. flexneri* [16], in the context of anti-bacterial activity, one of the key distinctions is the presence of a Type VI Secretion System (T6SS). A T6SS has been identified in *S. sonnei* but only remnants remain in *S. flexneri* (16). In one clinical isolate it has been demonstrated that this T6SS plays a key role in outcompeting *S. flexneri* during infection or in polymicrobial environments thus allowing *S. sonnei* to gain a competitive advantage [17].

The T6SS is a proteinaceous molecular machinery that is capable of delivering effector proteins directly to adjacent cells, both to the host and other microbes [18]. The T6SS has been found in approximately 25 % of all Gram-negative bacteria [19]. It is one of many mechanisms that allow bacteria to gain a competitive advantage within complex polymicrobial communities where habitat and resources are limited. Recent discoveries have shown that the T6SS is capable of mediating interbacterial interactions [18, 20], targeting host cells during infection [21, 22], targeting eukaryotic competitors such as yeast [23] and even to play a role in the scavenging of metal ions [24].

The assembly and multi-protein structure of the T6SS is well conserved across the Proteobacteria, but there are large variations in their regulation [20, 25–27] and effector repertoire [28, 29]. The minimum functionality of the T6SS requires 13 sub-units (TssA-TssM), which are sometimes referred to as the core T6SS genes [30, 31]. Secreted T6SS effector proteins are genetically encoded with cognate immunity proteins which prevent self-intoxication and toxicity to kin-bacteria [28, 29, 32]. A recent study investigating the anti-bacterial activity of *S. sonnei* using bacterial genome wide association studies combined with high throughput phenotyping indicated that the ability of *S. sonnei* to kill *E. coli* had no association with the T6SS [33].

An additional anti-bacterial mechanism common to *S. sonnei* but not *S. flexneri* is the expression of E-class colicins from a small multi-copy plasmid, spB. Colicins were named due to their first characterisation in *E. coli*, with later discoveries classifying colicins into sub-groups based on their import mechanism [34]. Further advances into bacterial-secreted compounds revealed that many other species of bacteria also encoded their own versions of colicins, collectively named bacteriocins. Colicins generally have a narrow target range due to target receptor specificity [34, 35], with E-class colicins utilising the vitamin B-12 receptor (BtuB) for uptake into target cells. Genetically, colicins are usually encoded on plasmids, with E-class colicins encoded on small high-copy number plasmids. These colicinogenic plasmids encode for the colicin, cognate immunity gene and colicin lysis protein. The majority of E-class colicins are nucleases except for colicin E1 which has a pore-forming active domain [34]. Colicin immunity proteins are produced to prevent self-intoxication and toxicity to sister bacteria. Unlike the T6SS, colicins are a collective anti-bacterial mechanism as they do not benefit the producer but rather cells of the same genotype.

In this study, we investigated the contribution of the anti-bacterial activity of a contemporary clinical isolate of *S. sonnei* (SS381) alongside a *S. sonnei* isolate (CIP106347) that was previously demonstrated to exhibit T6SS-mediated anti-bacterial activity [17]. In our experiments the anti-bacterial activity was derived from the carriage of E-class colicins encoded on the plasmid, spB, rather than from the T6SS. We also provide evidence that the *S. sonnei* T6SS is in the process of functional degradation with multiple mutations inactivating key components.

## Results

### *S. sonnei* has potent anti-bacterial activity *in vitro* against *E. coli* that is not due to T6SS activity

**Fig. 1.**
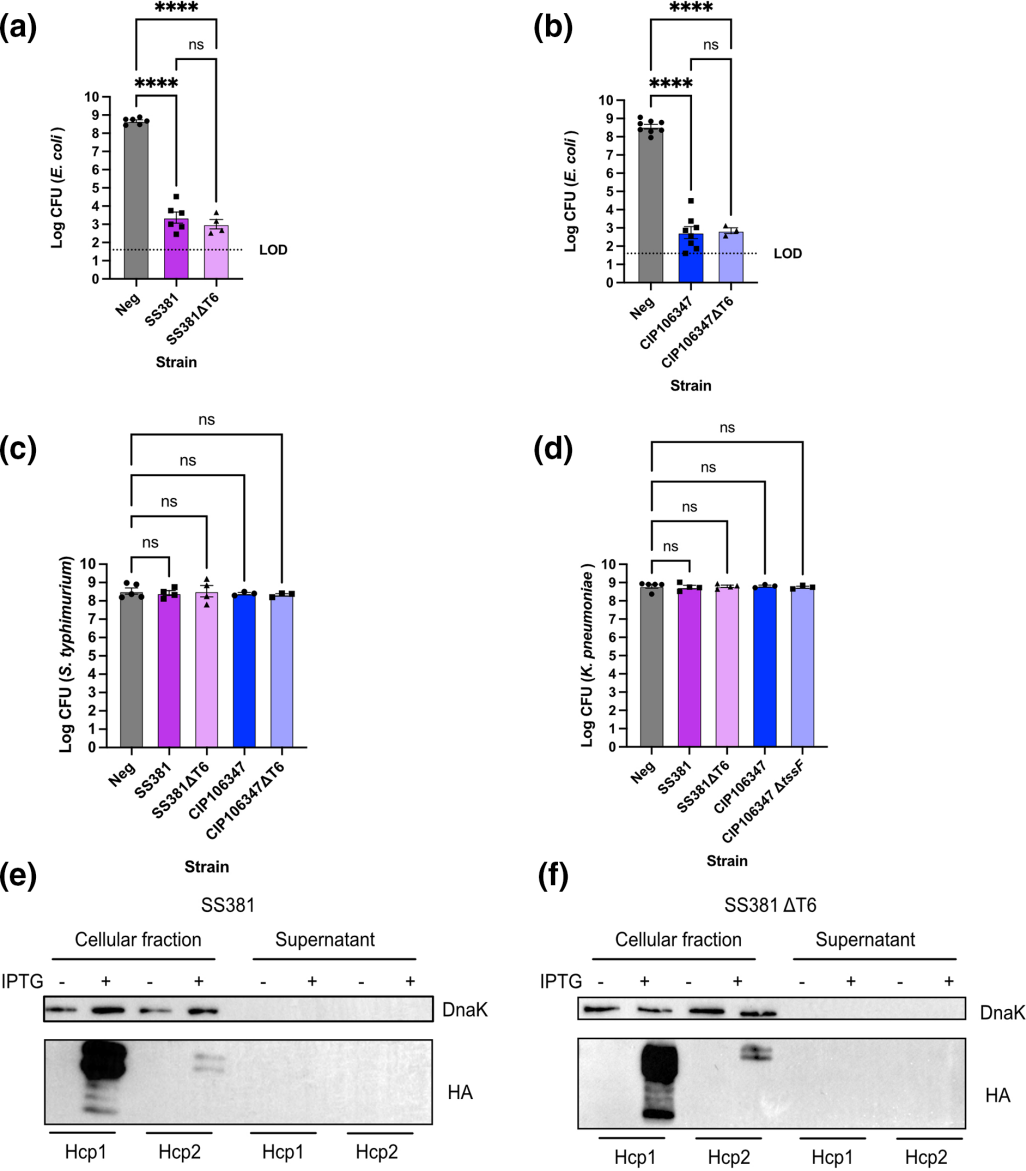
*S. sonnei* has potent anti-bacterial activity against *E. coli* but not *S. typhimurium* or *K. pneumoniae*. (a and b) Competition assays of *S. sonnei* and *E. coli* were conducted to assess the anti-bacterial activity of *S. sonnei*. Two representative isolates a) SS381 and b) CIP106347 and their isogenic T6SS mutants were competed against *E. coli* BZB1011-Km (a) or *E. coli* BZB1011 (b). The two *S. sonnei* isolates were also competed against c) *S. typhimurium* SL1344 d) and *K. pneumoniae* 43 816. The amount of *E. coli*, *S. typhimurium* and *K. pneumoniae* remaining after a competition with *S. sonnei* was enumerated to measure the anti-bacterial activity of *S. sonnei*. Each graph represents the mean±SEM of at least three biological replicates. A one-way ANOVA with Tukey’s correction was applied. **** = *p*<0.00001, ns=non-significant. LOD=Limit of detection. (e and f) Inducible Hcp1 and Hcp2 with C-terminal HA fusions were expressed from plasmids in (e) SS381 and (f) SS381ΔT6. Following Hcp induction, bacteria were centrifuged to separate the secreted fraction (supernatant) and the cellular fraction. Proteins were then separated by SDS-PAGE. The cytoplasmic protein DnaK was used as a loading control for the cellular faction. Hcp-HA fusion proteins were detected in all induced cellular factions but not the supernatant factions.

To investigate the contribution of the *S. sonnei* T6SS during competition with competitor bacteria, two representative isolates of *S. sonnei* were competed against *E. coli*, *S. typhimurium* or *K. pneumoniae*. T6SS competition assays were conducted on agar plates to ensure bacteria were in physical contact with each other as the T6SS is a contact-dependent secretion system. Isogenic *S. sonnei* T6SS-defective mutants were made by deleting a core component of the T6SS, *ΔtssF*. Both wild-type *S. sonnei* strains displayed potent anti-bacterial activity against *E. coli* ((Fig. 1a, b) but not against *S. typhimurium* (Fig. 1c) or *K. pneumoniae* (Fig. 1d). Unexpectedly, the T6SS mutants (ΔT6) for both *S. sonnei* strains displayed no statistically significant difference compared to the wild-type strains in their ability to inhibit the growth of *E. coli* (Fig. 1a, b). This suggests that the anti-bacterial activity measured in these assays is not due to T6SS activity.

As not all T6SS have anti-bacterial activity, Hcp secretion was also measured. Hcp forms the tube of the T6SS and is released into the extracellular milieu during active T6SS secretion [36]. Two genes encoding Hcp were identified within the *S. sonnei* T6SS operons and both were cloned and expressed as IPTG-inducible HA-fusion proteins. Secreted (supernatant) and non-secreted (cellular) fractions were separated and probed by immunoblot for Hcp presence. Hcp1 and Hcp2 HA-fusion proteins were detected in the cellular factions for both wild-type and *ΔtssF* strains upon induction with IPTG (Fig. 1e, f). However, there was no detection of Hcp HA-fusion proteins in any of the supernatant fractions indicating a lack of T6SS secretion activity. DnaK was used as a control to demonstrate there was no contamination of cytoplasmic proteins in the supernatant fraction.

### Contemporary *S. sonnei* have acquired mutations within the T6SS and are therefore non-functional T6SS

**Fig. 2.**
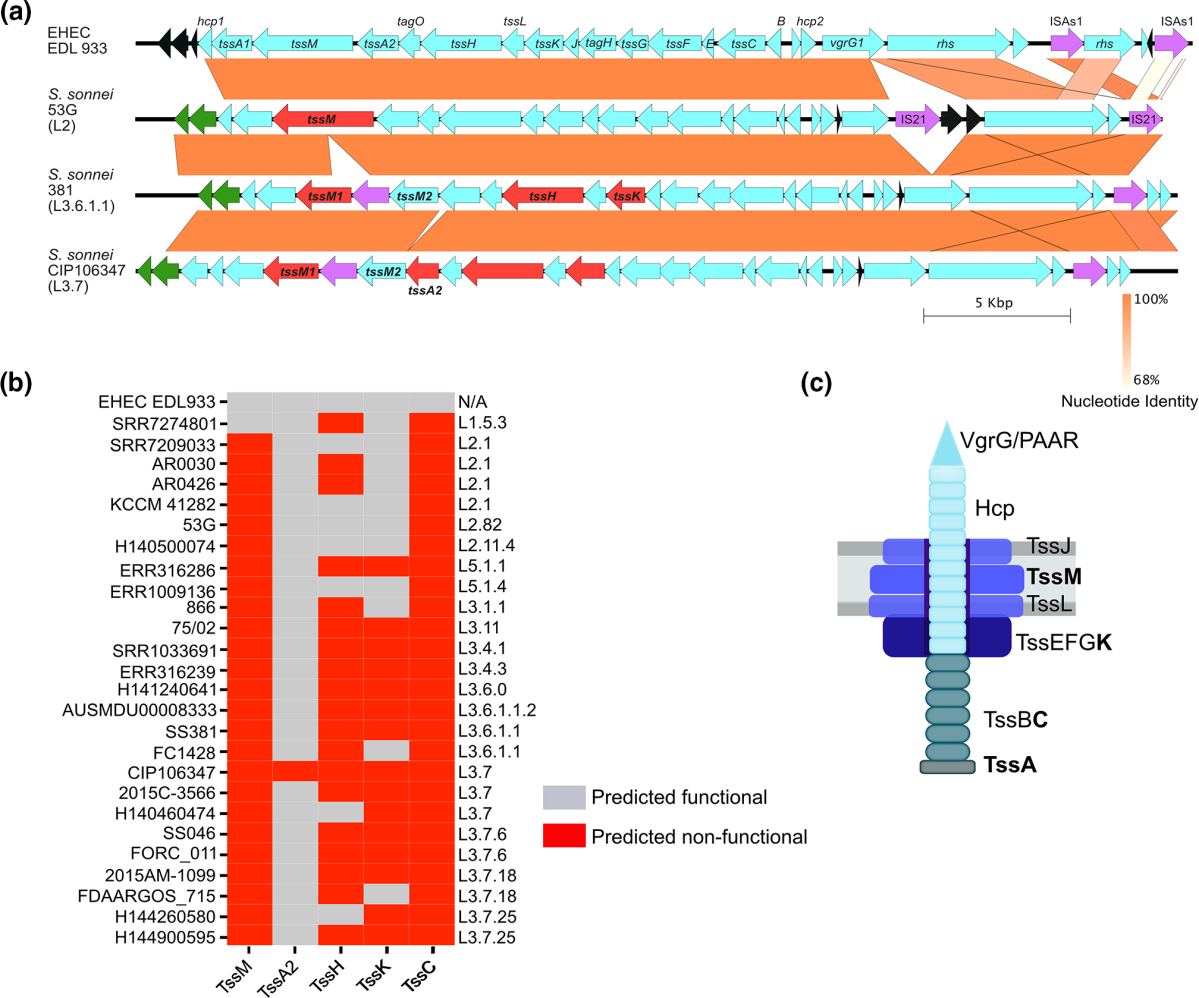
An *in silico* analysis of the T6SS cluster across three isolates of *S. sonnei* reveal mutations within the T6SS operon. (**a**) The T6SS operons were identified and aligned using BlastN and visualised in EasyFig. The reference genome in this synteny analysis was the T6SS operon from EHEC EDL933. Nucleotide percentage identity is denoted by the strength of the orange shading as indicated by the legend in the bottom right. EHEC EDL933 has a T6SS operon that is very similar to the published *S. sonnei* T6SS operon and has been shown to be functionally active. Core genes are labelled for EDL933 and *S. sonnei* 53G. Genes predicted to be non-functional are denoted in red. Genes of unknown function or unable to be annotated are coloured in black. Insertion sequence elements or transposons are coloured in purple. TssC is annotated as TssC1 (182 bp) and 2 (296 bp). (**b**) *In silico* analysis of 20 *S*. *sonnei* T6SS clusters reveal predicted non-functional proteins in red and predicted functional proteins in grey. The strain name is labelled on the left of the graph and the genotype of each isolate is indicated on the right of the graph. The non-functional status of TssM is acquired through different mutations; 53G, AR0426, KCCM 41282 have a point mutation leading to a premature stop codon while all others have an IS integrated. (**c**) A graphical representation of the *S. sonnei* T6SS with predicted non-functional components highlighted in bold.

Given the recent study indicating the ability of *S. sonnei* to kill *E. coli* had no association with the T6SS [33], we decided to investigate the T6SS in more detail. The T6SS gene clusters from a selection of strains were identified and compared: EHEC EDL933 which contains a T6SS closely related to *S. sonnei* [18] that has been demonstrated to be functional [21], *S. sonnei* 53G (a well-studied isolate of lineage two), SS381 and CIP106347 as the representative isolates from lineage three (Fig. 2a). This analysis illustrated that the *S. sonnei* T6SS is analogous to the EHEC EDL933 T6SS as predicted through sequence homology. However, a more detailed analysis revealed that several T6SS core genes in *S. sonnei* appear to be non-functional when translated (ORFs coloured in red, Fig. 2a). This is due to indels leading to a frameshift and premature stop codon, or the presence of an insertion sequence in the case of *tssM* for SS381 and CIP106347. We then searched SS381 and CIP106347 genomes for paralogs of TssM, TssA2, TssH (ClpV), TssK and TssC. TssH from SS381 and CIP106347 have 33.64 % and 35.04 % homology respectively to the ClpB ATPase. *Francisella tularensis* has been reported to use ClpB instead of TssH for a functional, but atypical T6SS [37] and therefore we cannot rule out that TssH can be complemented by ClpB in these *S. sonnei* strains. No paralogs were identified for TssM, TssA2, TssK or TssC.

To investigate the predicted functionality of the T6SS further, a larger set of *S. sonnei* WGS were extracted from the NCBI database to examine their T6SS locus. This collection of 23 additional *S. sonnei* isolates (accession numbers available in Table 1) was examined to investigate the prevalence and variety of functional and non-functional T6SS core components. The analysis showed that among the *S. sonnei* isolates, the T6SS core components; TssM, TssH, TssK and TssC were routinely predicted to be non-functional. A summary heatmap is shown in Fig. 2b. Of note, lineage two *S*. *sonnei* isolates 53G, AR0030, AR0426 and KCCM 41282 have a single TssM which is predicted non-functional by the presence of an internal stop codon. Lineage three isolates in this dataset have a TssM which is predicted non-functional due to an insertion sequence in the gene, annotated as TssM1 and 2. This genomic data provides further evidence that the *S. sonnei* T6SS may not be active due to mutations within multiple core components as illustrated in Fig. 2c.

**Table 1.** Bacterial accession numbers used in *in silico* analysis

Strain name	Accession no.	S. sonnei genotype [49]
EHEC EDL933	NC_002655.2	n/a
S. sonnei SRR7274801	SRR7274801	1.5.3
S. sonnei SRR7209033	SRR7209033	2.1
S. sonnei AR-0030	NZ_CP032523.1	2.1
S. sonnei AR-0426	NZ_CP044151.1	2.1
S. sonnei KCCM 41282	NZ_CP041511	2.1
S. sonnei 53G	HE616528.1	2.8.2
S. sonnei H140500074 (SS074)	SRR5034528	2.11.4
S. sonnei ERR316286	ERR316286	5.1.1
S. sonnei ERR1009136	ERR1009136	5.1.4
S. sonnei 866	NZ_CP022672.1	3.1.1
S. sonnei 75/02	NZ_CP019689.1	3.1.1
S. sonnei SRR1033691	SRR1033691	3.4.1
S. sonnei ERR316239	ERR316239	3.4.3
S. sonnei H141240641 (SS641)	SRR5034532	3.6.0
S. sonnei H140860381 (SS381)	SRR10996750	3.6.1.1
S. sonnei FC1428	NZ_CP041322.1	3.6.1.1
AUSMDU00008333	NZ_LR213458.1	3.6.1.1.2
S. sonnei CIP106347	GCF_025908455.1 [33]	3.7
S. sonnei 2015C-3566	NZ_CP022457.1	3.7
S. sonnei H140460474 (SS474)	SRR5034518	3.7
S. sonnei 2015AM-1099	NZ_CP021144.1	3.7.18
FDAARGOS_715	NZ_CP046286.1	3.7.18
S. sonnei H144900595 (SS595)	SRR4195720	3.7.25
S. sonnei H144260580 (SS580)	SRR 4195771	3.7.25
S. sonnei SS046	NC_007384.1	3.7.6
S. sonnei FORC_011	NZ_CP010829.1	3.7.6

### The *S. sonnei* T6SS is transcribed *in vitro*

**Fig. 3.**
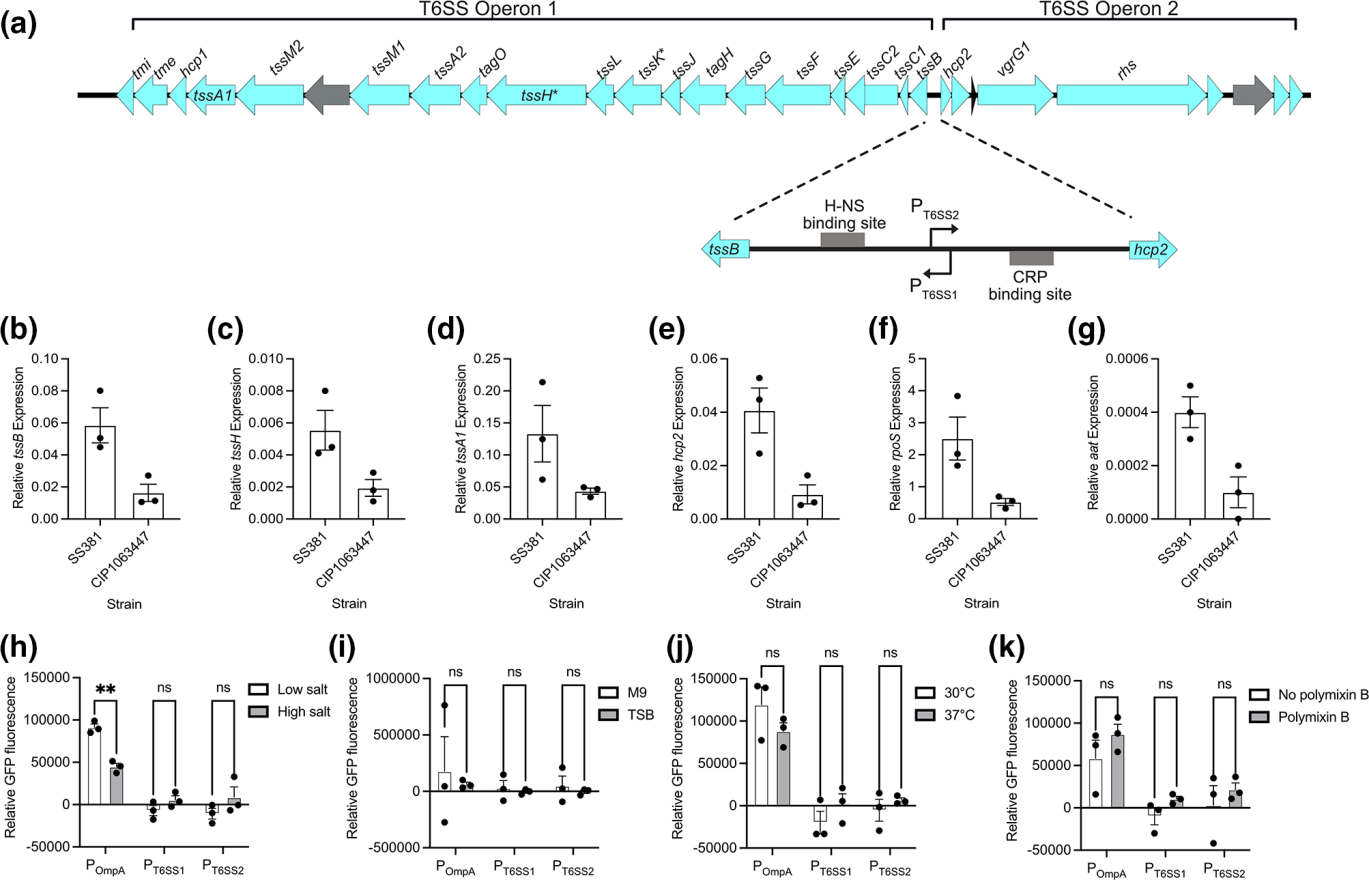
The *S. sonnei* T6SS is expressed at very low levels when under the control of its native promoters. (**a**) *In silico* analysis of the T6SS operon in *S. sonnei* reveals a bi-directional regulatory region. For both promoters the predicted transcriptional start sites (TSS) is indicated with an arrow. Putative binding sites for transcriptional regulators, H-NS and CRP identified by BProm are also indicated. (**b-e**) qRT-PCR was performed to determine the expression level of genes within both operons. Relative gene expression was determined by normalisation to *rhoB* as the bacterial house-keeping gene. Expression of (b) *tssB*, (**c**) *tssH* and (d) *tssA1* from T6SS operon 1 and (e) *hcp2* from T6SS operon 2 were quantified. For comparison highly expressed *rpoS* (f) and the lowly expressed *aat* (g) were measured. (**h-k**) The intergenic region between the two operons was inserted in both orientations into a promoter-less GFP vector to create a reporter construct to determine promoter activity. The promoter of *ompA* was used as a positive control. Transcriptional activity was measured by determining the relative fluorescence compared to bacteria without a reporter construct. Conditions tested included: (h) osmolarity, by culturing in low [8 mM] or high [595 mM] salt concentrations; (**i**) nutrient availability, by culturing in low (M9 minimal media) and high (TSB) nutrient conditions; (**j**) temperature, by culturing at 30 °C or 37 °C; and (k) membrane stress and permeability by adding polymyxin B (10 µg ml^−1^). A two-way ANOVA with Sidak’s correction was applied to test for significance. **= *p*<0.01, NS=non-significant.

Despite the mutations observed within components of the T6SS it is still possible the T6SS remains functional but has not been activated in our *in vitro* conditions. T6SS activity is often tightly regulated at a transcriptional level [26], we therefore investigated the region between the two operons in more detail. *In silico* prediction of this region indicated two predicted promoters controlling transcription of both operons with potential H-NS and CRP binding sites (Fig. 3a). The closest homolog of the *S. sonnei* T6SS operon in EHEC EDL933 was experimentally validated to be regulated by H-NS [21].

We used qRT-PCR to determine whether the T6SS operons were transcribed *in vitro* (Fig. 3b–g). We observed expression for all the genes tested (*tssB, tssH and tssA1* from operon 1 and *hcp2* from operon 2). We used two additional genes to compare the expression level: *rpoS* for high and *aat* for low expression respectively [38, 39]. This indicated that there was expression, albeit at a low level for both operons.

To determine if the level of transcription could be increased under different conditions, we constructed fluorescent T6SS reporter plasmids using the promoter regions of both operons to drive GFP expression. The OmpA promoter region was used as a control [40]. These three constructs were then tested in conditions that should relieve H-NS or CRP repression (temperature and glucose availability, respectively) or mimic T6SS attack (polymyxin B treatment) [41] (Fig. 3h–k). However, we saw no increased GFP expression under these conditions and therefore could not identify conditions to increase T6SS activity *in vitro*.

**Fig. 4.**
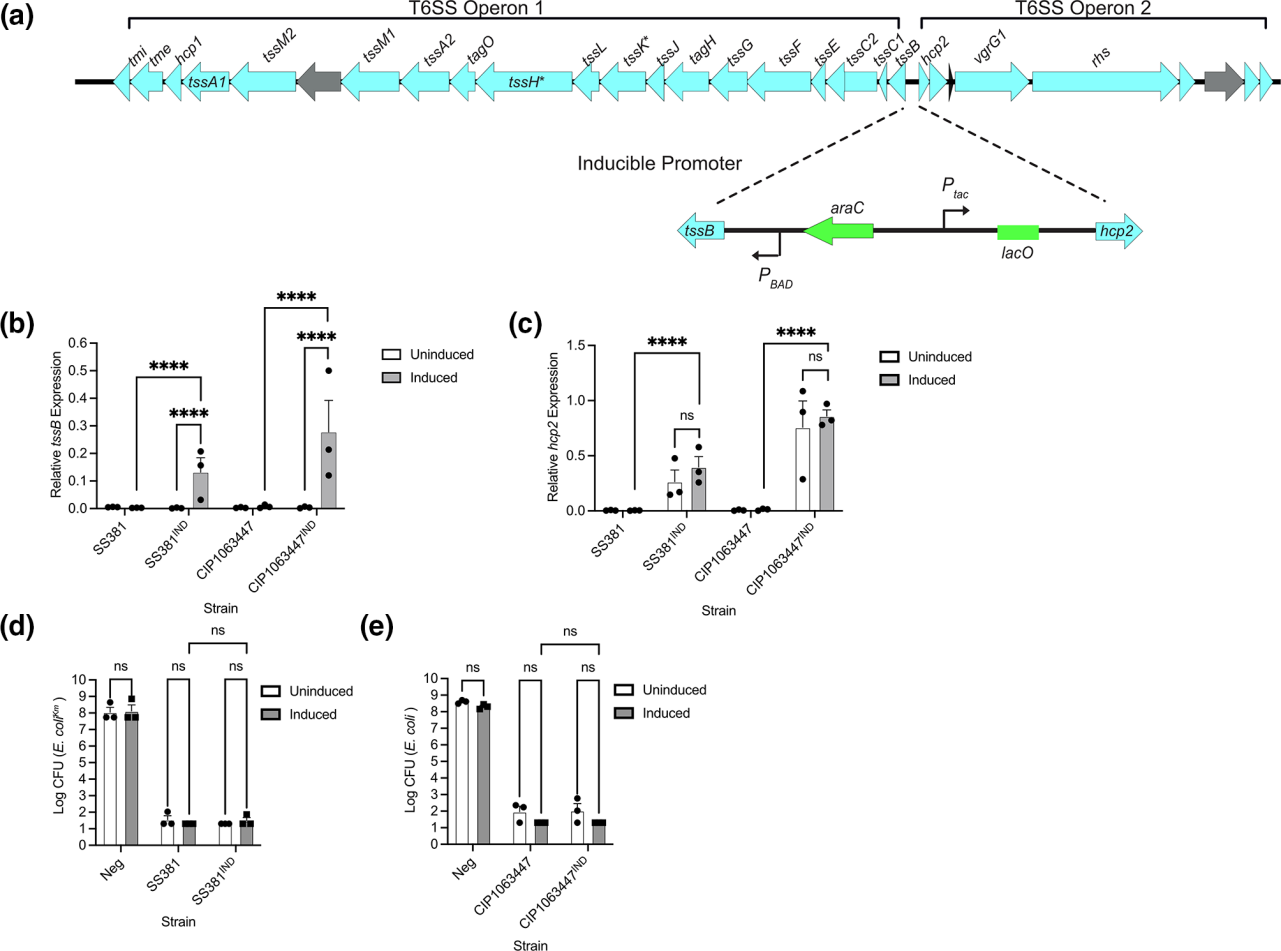
Replacement of the native promoters of the T6SS with an inducible version increases T6SS gene expression but does not show evidence of increased anti-bacterial activity. (**a**) A divergent promoter containing the P_BAD_ and P_tac_ was inserted to replace the native promoters within the regulatory region of the *S. sonnei* T6SS locus. The inducible promoters allowed for regulated control via the supplementation of arabinose and IPTG during bacterial culture. (**b-c**) RT-qPCR experiments were conducted using extracted RNA to determine gene expression of (b) *tssB* and c) *hcp2*. (**d-e**) The wild-type and inducible strains of *S. sonnei* were then utilised in competition assays with *E. coli*. The amount of *E. coli*, remaining after competition was enumerated to measure the anti-bacterial activity of *S. sonnei*. Two-way ANOVAs were performed with uncorrected Fisher’s LSD. ****= *p*<0.0001, ns=non-significant.

To activate the operon, we therefore chose to artificially induce T6SS transcription with a divergent promoter in which the two operons can be induced with arabinose and IPTG [42] (Fig. 4a). We confirmed transcription of both operons by qRT-PCR of the first gene in each operon (*tssB* and *hcp2* respectively, Fig. 4b, c). The inducible strains, SS381^IND^ and CIP1063447^IND^, showed arabinose-dependent expression of *tssB* from P_BAD_. However, *hcp2* was expressed even in the absence of IPTG, suggesting leaky expression from P_tac_. Importantly induction of SS381^IND^ and CIP1063447^IND^ expressed significantly more *tssB* and *hcp2* in comparison to the strains with the native promoters.

These strains were then used in bacterial killing assays (Fig. 4d, e) to determine whether increased expression of the T6SS would alter the anti-bacterial activity of *S. sonnei*. As previously observed, *E. coli* was efficiently killed by *S. sonnei* regardless of induction state or the absence/presence of the inducible promoter (Fig. 4d, e).

### *S. sonnei* anti-bacterial activity is mediated by colicins

**Fig. 5.**
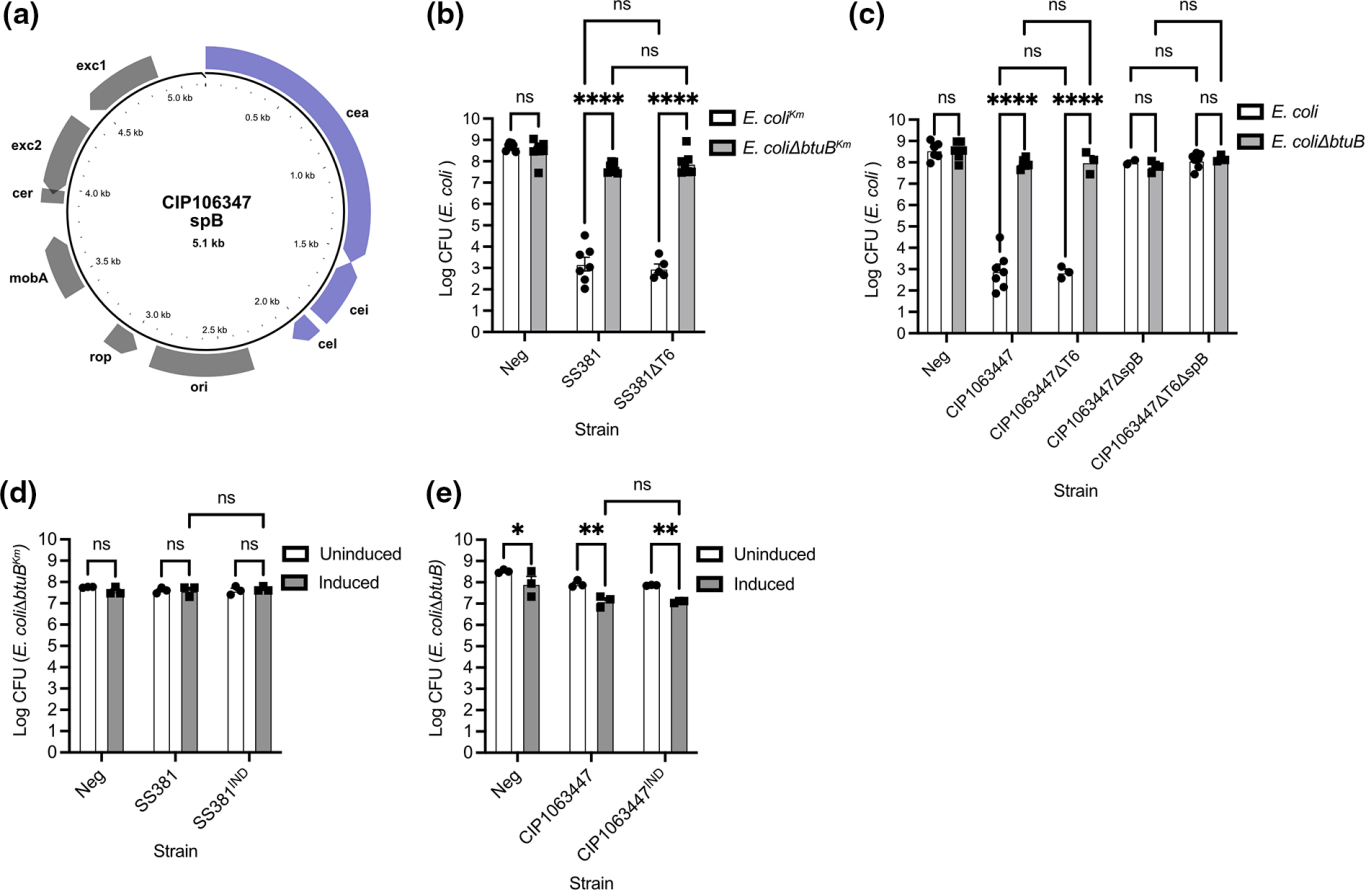
The anti-bacterial activity of *S. sonnei* is due to the activity of E-type colicins encoded on spB. (**a**) A plasmid map of CIP106347 spB depicting the colicin genes encoded: *cea* (the colicin gene), *cei* (the colicin immunity gene) and *cel* (colicin lysis gene required for colicin release). Also depicted are regions involved in replication (ori and *rop*), recombination (cer) and plasmid mobilisation genes (*mobA*, *exc2* and *exc1*). (**b-e**) Competition assays of an *E. coli* prey strain against attacker strains of *S. sonnei* were conducted at 37 °C during their exponential growth phases on agar plates. (**b and c**) The colicin-susceptible *E. coli* (or *E. coli^Km^*) but not the colicin-resistant *E. coli ΔbtuB* (or *E. coli ΔbtuB^Km^*) were efficiently killed by both SS381 and SS381ΔT6 (b) and CIP106347 and CIP106347ΔT6 (c). In addition, the anti-bacterial activity of CIP106347 was significantly reduced when the plasmid encoding spB was removed from CIP106347 (ΔspB and ΔspBΔT6). (**d and e**) The wild-type and inducible strains of *S. sonnei* were then utilised in competition assays with colicin resistant *E. coli ΔbtuB* (or *E. coli ΔbtuB^Km^*). The amount of *E. coli ΔbtuB* remaining after competition was enumerated to measure the anti-bacterial activity of *S. sonnei*. Each graph represents the mean±SEM of at least three biological replicates. Mixed-effects models (**b and c**) or two-way ANOVAs were performed with uncorrected Fisher’s LSD (**d and e**). *= *p*<0.05, **= *p*<0.01, **** = *p*<0.00001, ns=non-significant.

Since the anti-bacterial activity displayed by *S. sonnei* against *E. coli* in the competition assays does not appear to be due to T6SS activity, we next investigated the role of colicins. *S. sonnei* strains often harbour colicins on a small multicopy plasmid, spB which encodes E-class colicins. The spB backbone consists of plasmid mobilisation genes (*mobA, exc1, exc2*) and genes essential for colicin expression; *cea* (the colicin gene), *cei* (the colicin immunity gene) and *cel* (colicin lysis gene required for colicin release) (Fig. 5a). In order to examine if the anti-bacterial activity we observed in Figs 1 and 4 was due to colicin activity we used two approaches: removal of the colicin plasmid from *Shigella* strains or using a colicin-resistant prey strain.

E-class colicins exert their activity on susceptible bacteria after uptake via the OM receptor, BtuB [35, 43]. By using an *E. coli ΔbtuB* strain, the effects of E-class activity should therefore be eliminated as they cannot enter the target cell. When *E. coli ΔbtuB* was used as the prey strain against SS381 and CIP106347 T6SS^+^ and T6SS^-^ strains (Fig. 5b, c), the majority of anti-bacterial activity from *S. sonnei* was eliminated. This confirmed the anti-bacterial killing required the presence of BtuB and was therefore likely to be due to colicin activity.

To remove the colicin plasmid we used a CRISPR system targeting the pColE1 ori to cure spB from CIP106347 and CIP106347ΔT6 [44], creating strains CIP106347ΔspB and CIP106347ΔspBΔT6. Interestingly we were unable to cure spB from strain SS381, which may be due to the different activity of the colicins encoded by CIP106347 and SS381 (a pore-forming or DNAse colicin respectively). Removal of spB and therefore colicin production abrogated the anti-bacterial activity of CIP106347 when competed against *E. coli* (Fig. 5c). This further indicated that the anti-bacterial activity being measured was due to colicin activity.

We then went on to test the inducible T6SS strains in bacterial killing assays using the colicin resistant *E. coli ΔbtuB* as the prey to determine whether any T6SS activity could now be detected. The colicin resistant *E. coli ΔbtuB* was not killed by either the native or inducible versions of SS381 confirming no T6SS activity was occurring (Fig. 5d). We did see a significant difference in *E. coli ΔbtuB* recovery when competed with the induced versus non-induced CIP106347^IND^ (Fig. 5e). However, this difference was also seen for the native CIP106347 and even the negative control (no competing bacteria). Therefore we conclude that the addition of arabinose and IPTG caused an unexplained small reduction in the recoverable *E. coli ΔbtuB* in this assay. Therefore this is no evidence of T6SS activity even following synthetic induction of the T6SS operons.

### Colicins produced by *S. sonnei* are active against closely related bacteria

**Fig. 6.**
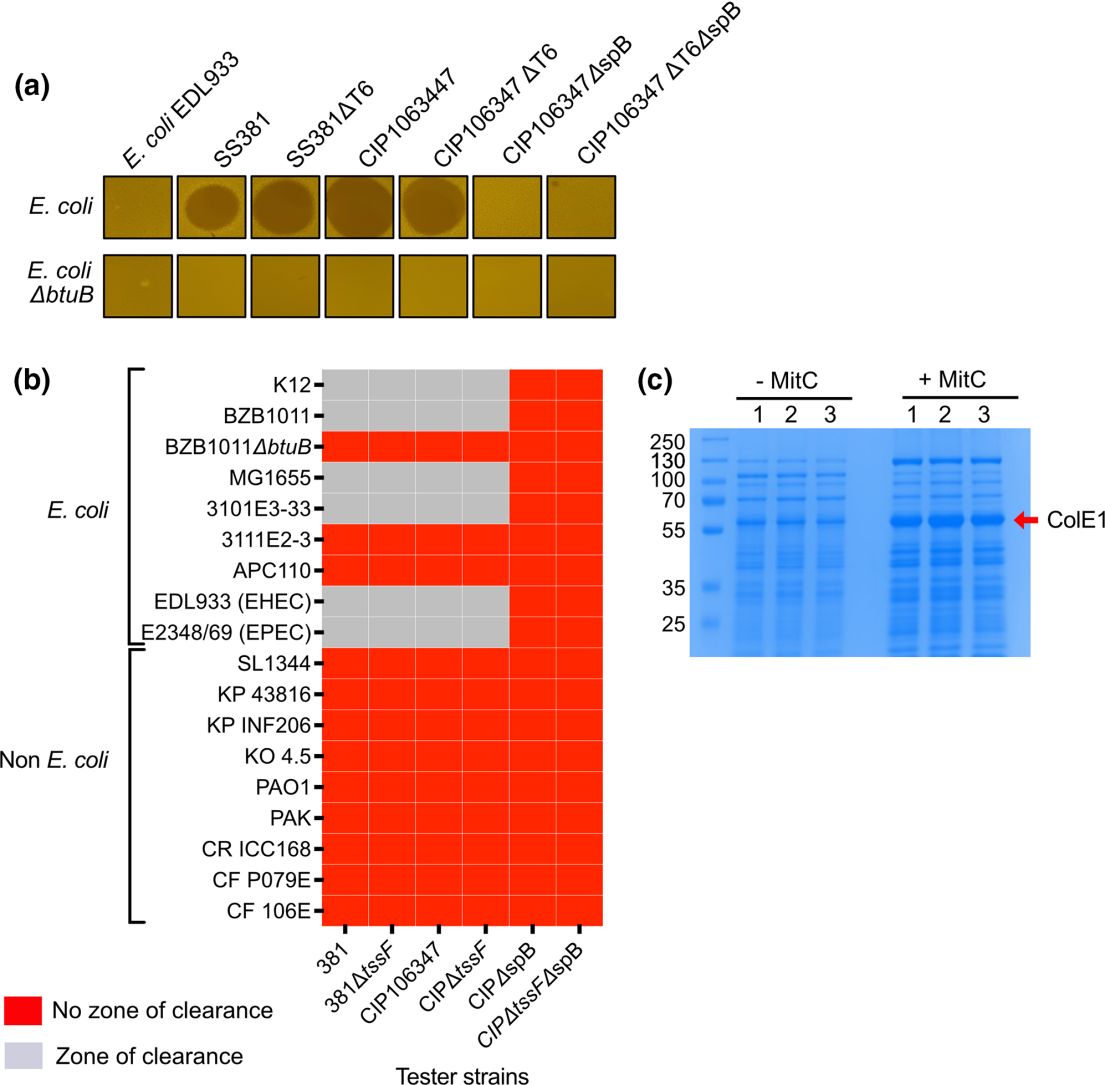
*S. sonnei* secreted colicins only target closely related bacteria and are dependent on the presence of BtuB**.** (a) A diffusible indicator assay where a susceptible *E. coli* or non-susceptible *E. coli ΔbtuB* are seeded in soft LB-agar. Filtered supernatants from the indicated strains were then spotted onto the *E. coli* lawns. Zones of clearance indicate the presence of a secreted colicin. (b) A panel of human associated commensal bacteria were also tested for their susceptibility to colicin. Zone of clearance (grey) indicates the presence of a secreted colicin and a sensitive strain. No zone of clearance (red) indicates either no colicin secreted or a resistant strain. Abbreviations; SL: *S. typhimurium,* KP: *K. pneumoniae,* KO: *K. oxytoca,* EC: *E. coli,* PA: *P. aeruginosa,* CR: *C. rodentium,* CF: *C. freundii.* (c) CIP106347 was grown in LB medium with or without the SOS agent Mitomycin C for 5h, pelleted, the supernatant filtered and concentrated. Concentrated *S. sonnei* supernatants were subjected to SDS-PAGE and proteins visualised by Coomassie blue staining. The predicted position of ColE1 (57.2 kDa) is indicated by the red arrow. MS analysis of this band indicated it was colicin E1. Molecular weight markers (in kDa) are indicated on the left.

To further test the anti-bacterial activity of colicins we measured the killing activity present in the supernatant of our isogenic mutants on *E. coli* and *E. coli ΔbtuB* using an overlay method (Fig. 6a). As predicted from the competition assays the supernatant of all strains except those cured of spB were able to kill wild-type *E. coli*, but not *E. coli ΔbtuB*. This indicates the presence of a secreted anti-bacterial agent encoded on spB that requires BtuB for activity.

Colicins target closely related bacteria [34, 35, 45, 46]. To test the specificity of the colicins produced by our clinical isolates we screened a panel of human-associated commensal bacteria, which also included some potential pathogens (Fig. 6b). The majority of *E. coli* isolates tested were susceptible to colicin-mediated killing, with only two wild-type isolates (a hospital isolate 3111E2-3 and a faecal isolate APC110) resistant to colicin mediated killing. All non-*E. coli* strains tested were resistant to colicin-mediated killing demonstrating the narrow activity spectrum of these colicins.

We used WGS data and PCR sequencing of the *cea* and *cei* genes to identify the colicins present in a selection of clinical isolates and identified the colicins encoded by these strains as E1 (CIP106347), E7 (SS381), E2 (SS595), E5 (SS474) and E7 (SS074). Of these colicins E1 is a pore-forming colicin while the remainder cleave either DNA (E2 and 7) or tRNA (E5). We confirmed the presence of colicin E1 in the supernatant of CIP106347 by mass spectrometry (MS). Colicin expression was upregulated by addition of mitomycin C and visualised by SDS-PAGE of the concentrated supernatants. A band at 57 kDa (MW of colicin E1) was excised and analysed by MS. The most abundant protein identified was Colicin E1 with 222 spectral counts in the no mitomycin C lane and 1055 counts in the presence of mitomycin C (Fig. 6c).

**Fig. 7.**
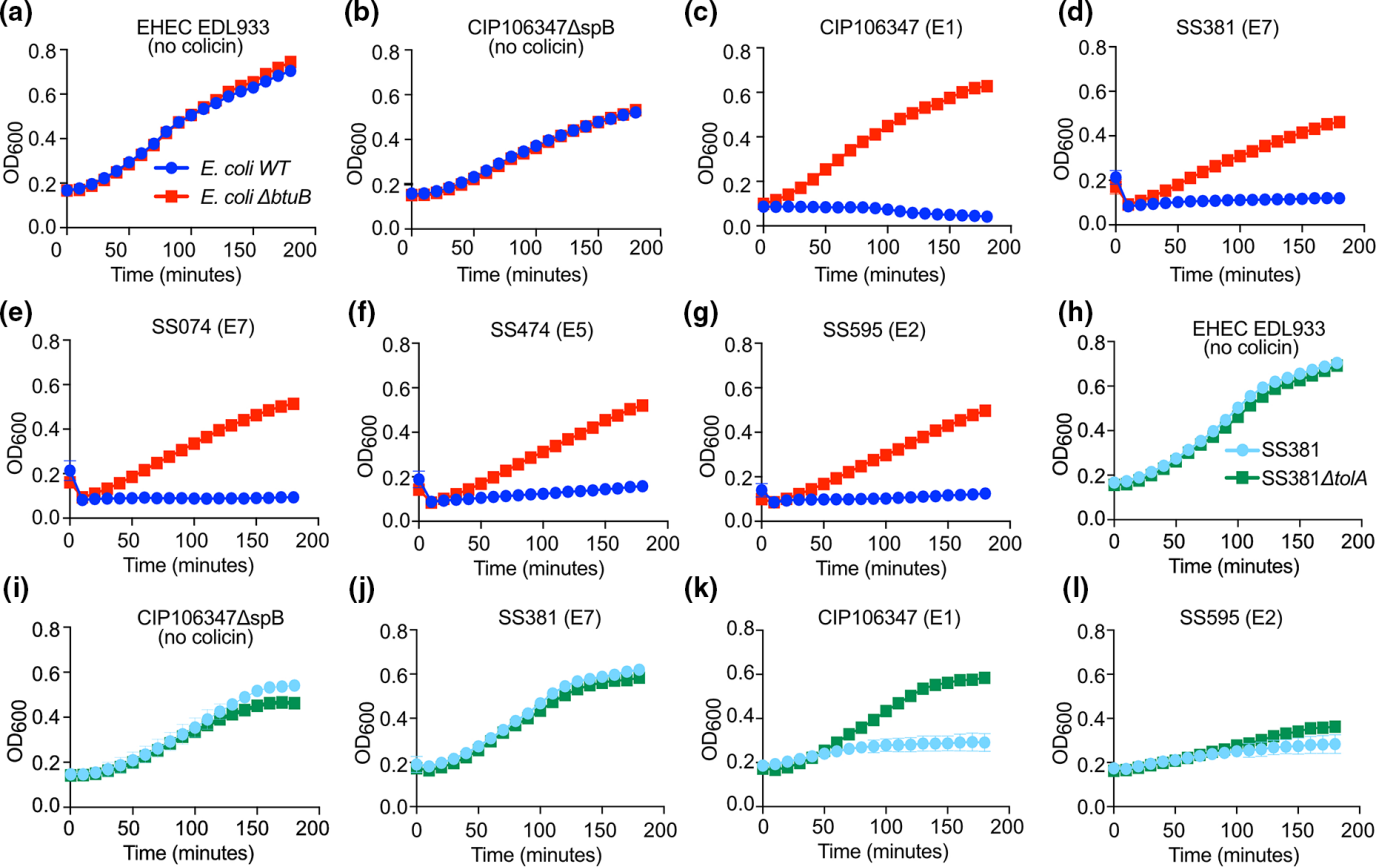
Colicin activity is dependent on BtuB and, for Colicin E1, TolA. Colicin-containing supernatant from the indicated strains of *S. sonnei* or EHEC was added to (**a-g**) *E. coli* WT and *E. coli ΔbtuB* or (**h-l**) SS381 and SS381*ΔtolA*. The OD600 of *E. coli* or *S. sonnei* was measured every ten minutes for 3 h while shaking at 37 °C. (**a-g**) Growth inhibition of the WT *E. coli* but not *E. coli ΔbtuB* was observed for all colicin-containing supernatants tested. (**h-l**) No growth inhibition of SS381 and SS381*ΔtolA* was seen for (h) EHEC control supernatant, (**i**) CIP106347ΔspB or (j) SS381 E7-containing supernatant. (**k**) Growth inhibition of the SS381 but not SS381*ΔtolA* was observed for CIP106347 E1-containing supernatant, while (l) both SS381 and SS381*ΔtolA* were inhibited by SS595 E2-containing supernatant. Each graph represents the mean±SEM of three biological replicates.

We then used our collection of *S. sonnei* strains encoding different colicins to investigate colicin killing using a quantitative plate reader assay (Fig. 7a–g). The supernatant of each strain was added to *E. coli* and *E. coli ΔbtuB* and the OD_600_ measured over a 3 h time course. In the presence of *E. coli* EDL933 supernatant, which contains no identifiable colicins, or CIP106347ΔspB both strains grew equally well (Fig. 7a, b). By contrast the supernatant of all *S. sonnei* strains restricted the growth of wild-type *E. coli,* but not *E. coli ΔbtuB* over the time course, suggesting the presence of E-type colicins in all tested *S. sonnei* supernatants (Fig. 7c–g).

E1 colicin activity has previously been demonstrated to be dependent on TolA for import into the prey bacteria, while other E-type colicins can use either TolA or TonB for import [34, 47]. We tested the ability of supernatants containing pore-forming (E1), and non-pore-forming (E2 and E7) to inhibit the growth of SS381 and SS381*ΔtolA* (Fig. 7h–l). The supernatants of EDL933 and CIP106347ΔspB were inactive against both SS381 strains as expected (Fig. 7h, i). The supernatant of SS381 (colicin E7) was also inactive against both SS381 strains due to the presence of the E7 immunity protein in this strain (Fig. 7j). As predicted the supernatant containing the pore-forming E1 colicin was able to inhibit the growth of SS381 but was unable to inhibit growth of SS381*ΔtolA* (Fig. 7k). The supernatant containing the E2 colicin was able to inhibit both SS381 and SS381*ΔtolA*, indicating TolA-independent import (Fig. 7l).

## Discussion

The discovery of a T6SS in *S. sonnei* but not in *S. flexneri* was predicted to favour *S. sonnei* during niche competition in the gut [17]. We were unable to demonstrate T6SS-dependent anti-bacterial killing by *S. sonnei* in our *in vitro* assays. Initially, we used isogenic T6SS^+^ and T6SS^-^ *S. sonnei* strains and found no difference in their anti-bacterial capability against *E. coli. E. coli* were very efficiently killed in this competition assay, however the effect was independent of the T6SS. The *S. sonnei* strains tested were unable to inhibit closely related Enterobacteriaceae such as *S. typhimurium* or *K. pneumoniae,* indicating a narrow spectrum of anti-bacterial activity. We then used a secretion assay to determine if the T6SS was active, but perhaps not involved in anti-bacterial competition. While Hcp expression was observed no Hcp was detected in the supernatant indicating there was no T6SS activity.

The lack of measurable T6SS activity led us to perform an *in silico* analysis of the T6SS from *S. sonnei* WGS. This showed that all T6SS clusters were predicted to be non-functional due to inactivation of at least one, but often multiple T6SS core gene across all lineages [48, 49]. While all strains had mutations in key T6SS components there is variability in the requirement for different components in different bacteria and it may be that some of these mutations do not disrupt the function of the T6SS. For example, the *C. rodentium* TssM is expressed as a truncated 807aa protein but translational slippage allows a 1129aa full length protein to also be translated. In fact, both forms of TssM are required for T6SS activity in *C. rodentium* [50]. As we had been unable to find a condition in which we could increase expression from the native T6SS promoters we instead chose to create an inducible T6SS to confirm whether the T6SS was functional despite the observed mutations. However, even this strain displayed no anti-bacterial activity suggesting that the accumulated mutations within the *S. sonnei* T6SS may render it non-functional.

One of the strains used in this study *S. sonnei* CIP106347 was previously demonstrated to possess T6SS-dependent anti-bacterial activity *in vitro* and *in vivo* against a lab isolate of *E. coli* and two isolates of *S. flexneri* [17]. Under the conditions we tested, we were unable to show T6SS-dependent anti-bacterial activity with this isolate. We cannot rule out that a threshold level of induction is required for activity to be observed which we did not reach even with our inducible strains. However, our data is in agreement with a recent computational-based study which indicated the ability of *S. sonnei* to kill *E. coli* was strongly associated with colicin genes rather than the T6SS [33].

We therefore sought to provide experimental data to show that the anti-bacterial activity that we observed for *S. sonnei* against *E. coli* was due to colicin activity [13]. By using both an *E. coli* strain that was resistant to E-class colicins (*ΔbtuB*) and *S. sonnei* isogenic strains with no colicins (ΔspB), we confirmed that the *S. sonnei* anti-bacterial activity measured in our assays was indeed due to the presence of colicins. We further demonstrate for colicin E1 from strain CIP106347 that it is upregulated by mitomycin C and is reliant on TolA for import into the recipient cell. Also in concordance with existing literature about colicins [34, 35, 45, 51, 52], we show that activity of *S. sonnei* colicins tested within this study is restricted to closely related bacteria, i.e. *E. coli* strains. It is of note that two out of eight of the *E. coli* isolates were resistant to colicin-mediated killing. These two strains were a human faecal isolate and a hospital isolate respectively. The ability of these strains to resist colicin killing could be due to a resistant BtuB phenotype or the presence of compatible colicin immunity genes.

To conclude, we have experimental evidence validating observations that *S. sonnei* anti-bacterial activity is associated with colicins rather than the T6SS [33]. We hypothesise that this anti-bacterial activity is likely one factor that helps *S. sonnei* establish itself during infection of the human gut by competing with closely related strains for common resources in the nutrient limited colon. *Shigella* species are human adapted pathogens and the primary replicative niche of *S. flexneri* is the nutrient-rich IEC cytosol. However, *S. sonnei* does not access the IEC cytosol as efficiently as *S. flexneri* [53] and therefore it may require additional mechanisms in order to compete with the resident microbiota for limited resources in the terminal ileum and colon. *S. sonnei* strains are closely related to *E. coli* [54] and are likely to directly compete with the resident *E. coli* commensals for nutrient availability in this niche. Our study suggests that *S. sonnei* may achieve this through the action of colicins.

## Methods

### Bacterial strains

Bacteria strains are described in Table 2. All *Shigella* strains were routinely cultured in Tryptic soy broth (TSB) or on Tryptic soy agar (TSA). TSA plates were supplemented with 0.01 % Congo red to identify and culture *Shigella* colonies that retained the large virulence plasmid. *Shigella* strains were maintained at 37 °C. Non-S*higella* strains were routinely maintained in Lysogeny Broth (LB) or on Lysogeny Agar (LA) unless otherwise stated. The following antibiotics were supplemented as required: ampicillin (Amp, 100 µg ml^−1^), kanamycin (Km, 50 µg ml^−1^), chloramphenicol (Cm, 30 µg ml^−1^), gentamicin (Gm, 20 µg ml^−1^), nalidixic acid (Nal, 50 µg ml^−1^). Where required for the inducible T6SS strains, liquid media was supplemented with 0.2 % (v/v) l-arabinose and 500 µM IPTG .

**Table 2.** Bacterial strains table

Strain name	Description	Origin
*S. sonnei* 53G	Lab isolate. (Lineage 2)	[59]
*S. sonnei* H140860381 (SS381)	Clinical isolate belonging to the global lineage III clade. Fluroquinolone resistant. (Lineage 3.6.1.1)	Claire Jenkins (UK Health Security Agency, UKHSA)
*S. sonnei* H140500074 (SS074)	Clinical isolate belonging to the Latin America lineage II-b. (Lineage 2.11)	Claire Jenkins (UKHSA)
*S. sonnei* H144900595 (SS474)	Clinical isolate belonging to the global lineage III clade. (Lineage 3.7)	Claire Jenkins (UKHSA)
*S. sonnei* H144900595 (SS595)	Clinical isolate belonging to the global lineage III clade. (Lineage 3.7.25)	Claire Jenkins (UKHSA)
SS381Δ*tolA*	SS381 TolA mutant deleted by homologous recombination.	This study
SS381ΔT6	T6SS mutant. *tssF* deleted by homologous recombination.	This study
SS381^IND^	SS381 derivative with native T6SS promoters replaced with pBAD and pTac.	This study
*S. sonnei* CIP106347 (CIP106347)	Clinical isolate. Sm^R^ and Tet^R^. (Lineage 3.7)	Kind gift from Marteyn Lab (University of Strasbourg) [17]
CIP106347ΔT6	T6SS mutant. *tssF* deleted by homologous recombination [17]	Kind gift from Marteyn Lab (University of Strasbourg)
CIP106347ΔspB	Colicin plasmid (spB) removed from parental strain CIP106347	This study
CIP106347ΔT6ΔspB	Colicin plasmid (spB) removed from CIP106347 Δ*tssF*	This study
CIP106347^IND^	CIP106347 derivative with native T6SS promoters replaced with pBAD and pTac.	This study
*E. coli* BZB1011 tn7-Pmax-GFP (*E. coli*)	W33110, Nal^R^, Str^R^	Kind gift from Elisa Granato (University of Oxford)
*E. coli* BZB1011 tn7-Pmax-GFPΔ*btuB* (*E. coli ΔbtuB*)	*btuB* deletion strain that renders this strain resistant to all E-class colicins	Kind gift from Elisa Granato (University of Oxford)
*E. coli* BZB1011 tn7-Pmax-GFP + pULTRA-GFP-Km (*E. coli* ^Km^)	W33110, Nal^R^, Str^R^, Km^R^	This study
*E. coli* BZB1011 tn7-Pmax-GFPΔ*btub*+pULTRA-GFP-Km (*E. coli ΔbtuB* ^Km^)	*btuB* deletion strain that renders this strain resistant to all E-class colicins. Nal^R^, Str^R^, Km^R^	This study
EHEC EDL933	Enterohemorrhagic *E. coli*. Human pathogen	[60]
EPEC E2348/69	Enteropathogenic *E. coli*. Human pathogen	[61]
*E. coli* 3101E3-33	Human commensal strain. Isolated from a human faecal sample from a Crohn’s patient.	Kind gift from Frankel Lab (Imperial College London)
*E. coli* 3111E2-3	Human commensal strain. Isolated from a human faecal sample from a Crohn’s patient.	Kind gift from Frankel Lab (Imperial College London)
*E. coli* APC110	Hospital isolate	Kind gift from Frankel Lab (Imperial College London)
*E. coli* K12	Non-pathogenic *E. coli*	
*E. coli* MG1655	Non-pathogenic *E. coli*	
*E. coli* DH5α	*E. coli* used for routine cloning.	
*E. coli* CC118λpir	pSEVA plasmid propagating strain	[55]
*E. coli* 1047 helper pRK2013	Helper strain to mobilise pSEVA-612S from CC118λpir to receiver strains	[62]
*Salmonella enterica typhimurium* SL1344	Wild-type	[63]
*Klebsiella oxytoca* KO4.5	Isolated from murine gastrointestinal tract	Kind gift from Frankel lab (Imperial College London)
*Klebsiella pneumoniae* 43 816	Wild-type	ATCC
*Klebsiella pneumoniae* INF206	Clinical isolate	Kind gift from Holt Lab (Monash University)
*Pseudomonas aeruginosa* PAK	A laboratory reference strain	Kind gift from Filloux Lab (Imperial College London)
*Citrobacter rodentium* ICC168	Mouse pathogen	Kind gift from Frankel Lab (Imperial College London)
*Citrobacter freundii* P079E	Human commensal opportunistic pathogen isolated from infant faeces.	[64]
*C. freundii* P106E	Human commensal opportunistic pathogen isolated from infant faeces.	[64]

### Molecular biology

All routine PCR cloning used Q5 DNA polymerase or OneTaq DNA polymerase (New England Biolabs, NEB) following manufacturer protocols. Primers are shown in Table 3 and plasmids in Table 4. Cloning constructs were generated using restriction digests and ligations or using Gibson Assembly as per the manufacturer’s protocols (all enzymes from NEB). Constructs were confirmed by sequencing (Eurofins Genomics). *S. sonnei* deletion mutants were made using a tri-parental conjugation and recombination method as previously described [55, 56]. In brief, the T6SS deletion mutant was constructed by cloning *tssF* ±500 bp into the vector pSEVA-612S. *tssF* was then removed by an inverse amplification of this vector to yield a plasmid only containing the flanking regions of *tssF*. This deletion vector was mobilised and conjugated into the parental *S. sonnei* strain to yield merodiploid clones. Activation of the inducible I-SceI from pACBSR removed merodiploids and deletion mutants were screened by PCR for confirmation (Table 3). Subsequent removal of pACBSR was achieved by serial passaging in liquid culture without antibiotic selection.

**Table 3.** Primers table

Primer name	Primer sequence (5’−3’)	Description
*tssF*-fw	TAGTCGACATCCACACCGCCGTTACGAAAACTGATGT	500 bp of homology and coding region of *tssF* for cloning into pSEVA-612S. With SalI and XbaI sites.
*tssF*-rv	CTATCTAGATACCAGGTTGCGCTAAGCGTGAGA	
*tssF-*inv-fw	TTGTCAGTCCACCCGGTAATGCTGGC	Inverse primers to remove the coding sequence of *tssF* from pSEVA-612S-*tssF*
*tssF-*inv-rv	ATGGACGGAAAGAATCGGGCAGCATC	
*tssF-*check fw	GAGGGTCGTTCTTCCGGTGTGC	Primers to check deletion of *tssF*
*tssF-*check rv	GGCCTGGATGCTGGCATGCAAT	
colE-replicon fw	CCGACAGGACTATAAAGATACC	Primers to check removal of plasmids with colE-like replicons
colE-replicon rv	CTCAAGACGATAGTTACCGG	
pRL128-inv-rv	GAAGCAGCTCCAGCCTACAC	Primers to remove KanR cassette from pRL128
pRL128-inv-fw	GAACTGCAGGTCGACGGATC	
pRL128-prom-fw	GTGTTGGCATTTGGCTGTTTCCTGTGTG	Primers to amplify inducible promoters from pRL128-*ΔkanR*
pRL128-prom-rv	TTTTGCTCATGGGTATGGAGAAACAGTAG	
T6-Operon1-fw	GGATTACCCTGTTATCCCTACGCCAGTCGTCGGCACCG	Primers to amplify the Rv promoter and 500 bp of homology for the T6SS (*tssB*)
T6-Operon1-rv	AAACAGCCAAATGCCAACACCGTGTTATATCTCCATCACTG	
T6-Operon2-fw	CTCCATACCCATGAGCAAAAAATTTGAAGG	Primers to amplify the Fw promoter and 500 bp of homology for the T6SS (*hcp2*)
T6-Operon2-rv	TAATTACCCTGTTATCCCTATATTTATTGCTCACCAGAC	
*hcp1*-exp fw	ATTCCCGGGATGGCAAATATAAGTTATTTATC	*hcp1* from 53G with XmaI and KpnI sites for cloning into pSA10-4XHA
*hcp1*-exp rv	CAGGGTACCCCTGTACACGATCCTGCC	
*hcp2*-exp fw	ATTCCCGGGATGCCAACACCGTGTTATATC	*hcp2* from 53G with XmaI and KpnI sites for cloning into pSA10-4XHA
*hcp2*-exp rv	CAGGGTACCCTGCTTCCAGTGGTGCGC	
GFP-T6SS-1 fw	CGTTAACAAGGAGTTTACAAATGCGTAAAGGCGAAGAGC	Promoter region from T6SS operon 1 (*tssB*) cloned into pULTRA-GFP for fluorescent reporter assay
GFP-T6SS-1 rv	TAACATGACGGATCACTTTTCAGAAATCATCCTTAGCGAAAGCTAAG	
GFP-T6SS-2 fw	TAACATGACGGATCACTTTTATGCGTAAAGGCGAAGAGC	Promoter region from T6SS operon 2 (*hcp2*) cloned into pULTRA-GFP for fluorescent reporter assay
GFP-T6SS-2 rv	CGTTAACAAGGAGTTTACAACAGAAATCATCCTTAGCGAAAGCTAAG	
GFP-*ompA* fw	ATGATAACGAGGCGCAAAAAATGCGTAAAGGCGAAGAGC	Promoter region for *ompA* cloned into pULTRA-GFP for fluorescent reporter assay
GFP-*ompA* rv	TCAGACAAGCCTCCGCAAGGCAGAAATCATCCTTAGCGAAAGC	
T6HR2 fw	GGATTACCCTGTTATCCCTACGCCAGTCGTCGGCACCG	Amplify 500 bp homology from T6SS operon 2
T6HR2 rv	AAACAGCCAAATGCCAACACCGTGTTATATCTCCATCACTG	
pInd fw	GTGTTGGCATTTGGCTGTTTCCTGTGTG	Amplify inducible promoter from pRL128ΔkanR
pInd rv	TTTTGCTCATGGGTATGGAGAAACAGTAG	
T6HR1 fw	CTCCATACCCATGAGCAAAAAATTTGAAGG	Amplify 500 bp homology from T6SS operon 1
T6HR1 rv	TAATTACCCTGTTATCCCTATATTTATTGCTCACCAGAC	
RT-qPCR *rho* fw	GTGATGGCGTACTGGAGATATT	qPCR primers for *rho*.
RT-qPCR *rho* rv	GGTTGAAACGGCGGATTTG	
RT-qPCR *tssB* fw	CTGAAGGGAAGCACAACAGA	qPCR primers for *tssB*.
RT-qPCR *tssB* rv	ACGCAGTTCCAGCAATTTATTC	
RT-qPCR *hcp2* fw	TGGTCAGACCCAGGGAAATA	qPCR primers for *hcp2*.
RT-qPCR *hcp2* rv	AATTCCTGCACCAGCATCTC	
RT-qPCR *tssF* fw	AAATGTATCTGCTCGGTACGG	qPCR primers for *tssF*.
RT-qPCR *tssF* rv	ATGTCTCCTGGCTTTCTGTG	
RT-qPCR *tssH* fw	ATGTGCTCAACCTGTTCTACC	qPCR primers for *tssH*.
RT-qPCR *tssH* rv	GGCATGCTCGACTATTACCTG	
RT-qPCR *tssA1* fw	GCCAGGACTTACATGCTTTTG	qPCR primers for *tssA1*.
RT-qPCR *tssA1* rv	CGTCAACAGAGACTCAATGGG	
RT-qPCR *rpoS* fw	AGCTTATGGGACAACTCACG	qPCR primers for *rpoS*.
RT-qPCR *rpoS* rv	CGCTTCTCAACATACGCAAC	
RT-qPCR *aat* fw	CCTCACAGAATACCAGAAGCG	qPCR primers for *aat*.
RT-qPCR *aat* rv	TCATGCCCACTCCATTGAAG	

**Table 4.** Plasmids table

Plasmid	Description	Origin
pSEVA612S	Integrative plasmid with ori R6K and oriT for conjugation. Used in tri-parental conjugation based cloning. GmR.	[55]
pSEVA612S-*tssF*	pSEVA-612S derivative with flanking and coding region of SS381-*tssF*.	This study
pSEVA612S-*ΔtssF*	pSEVA-612S derivative constructed with inverse PCR of parental plasmid, used for genomic deletion of *tssF*.	This study
pACBSR	Expresses I-SceI and lambda-red induced by l-Ara used in tri-parental conjugation based cloning.	[65]
pFREE	Inducible CRISPR-Cas9 plasmid that removes ColE-like replicons. AmpRs	[44], pFREE was a gift from Morten Norholm (Addgene plasmid # 92 050
pRL128	Plasmid with pBAD and pTac in divergent orientations.	[42], pRL128 was a gift from Eric Cascales (Addgene plasmid # 40 180
pRL128*ΔkanR*	pRL128 derivative with kanamycin resistance cassette removed	This study
pJET-T6^IND^	pJET1.2 with promoter region from pRL128-ΔkanR and flanking T6SS operon 1 and 2 homology regions	This study
pSEVA612S-T6^IND^	pSEVA612S derivative with promoter region from pRL128-*ΔkanR* and flanking T6SS Operon 1 and 2 homology regions. Used to replace the native promoters by tri-parental conjugation based cloning	This study
pSA10-4XHA	IPTG inducible protein over-expression plasmid with integrated 4xHA tags	[66]
pSA10-4XHA-*hcp1*	pSA10 derivative with SS53G-hcp1	This study
pSA10-4XHA-*hcp2*	pSA10 derivative with SS53G-hcp2	This study
pULTRA-gfp-P_T6SS1_	Fluorescent reporter plasmid containing intergenic region of the *S. sonnei* T6SS, orientated to transcribe operon 1	This study
pULTRA-gfp-P_T6SS2_	Fluorescent reporter plasmid containing intergenic region of the *S. sonnei* T6SS, orientated to transcribe operon 2	This study
pUTRA-gfp-P*_ompA_*	Fluorescent reporter plasmid containing promoter region for *S. sonnei ompA*	This study
pULTRA-GFP-Km	Fluorescent reporter plasmid with a kanamycin resistance cassette used for selective plating	[67]

KanR was removed from pRL128 by inverse PCR using primers pRL128inv fw and rv. Then 500 bp flanking the intergenic T6SS promoter region, and the promoter region of pRL128ΔkanR were amplified with primers T6HR1 fw and rv, T6HR2 fw and rv and pInd fw and rv respectively. Fragments were Gibson assembled and cloned into pJET1.2 to form pJET-T6^IND^. The inducible promoter with flanking homology regions was digested from pJET-T6^IND^ with I-SceI and ligated into I-SceI digested pSEVA612S to form pSEVA-T6^IND^ which was then used in a tri-parental conjugation and recombination as above.

The removal of the small *S. sonnei* plasmid encoding colicin (spB) was achieved using the pFREE plasmid curing system (Table 4) [44].

Protein expression constructs for Hcp1 and Hcp2 (Table 3) were amplified from *S. sonnei* 53G and cloned into the pSA10-4xHA vector to add C-terminal HA tags to Hcp1 and Hcp2. These constructs were transformed into *S. sonnei* strains.

### RT-qPCR analysis of the T6SS

RNA was prepared from bacterial strains that were grown to exponential state as per manufacturer’s instructions (Qiagen RNeasy). cDNA was synthesised from the extracted RNA by following the manufacturer’s instructions (Promega, M-MLV Reverse Transcriptase). Samples were then probed with the appropriate primer sets with PowerUp SYBR Green (Thermofisher) in a Quantstudio 1 Real-Time PCR system (Thermofisher). Samples were compared by relative gene fold expression normalised to the housekeeping gene (*rhoB*) with the following formulas:

ΔCt=Ct (gene of interest)−Ct (housekeeping gene,(rhoB))

$$Relativegeneexpression=2^{-∆Ct}$$

### T6SS Hcp secretion assay

*S. sonnei* cultures were induced with 1 mM IPTG at OD_600_=0.2. After a further 60 min of growth, liquid cultures were harvested and concentrated to an OD_600_=5.0. The cell pellet (cellular fraction) was resuspended in reducing Laemmli buffer and heated at 95 °C for 5 min. The supernatant fraction was filter sterilised with a 0.22 µm filter and precipitated with trichloroacetic acid. The precipitated supernatant faction was resuspended in reducing Laemmli buffer and heated at 95 °C for 5 min. All samples were separated on a 15 % SDS-PAGE and proteins transferred to PVDF membranes. Membranes were probed with mouse α-HA (Biolegend) or mouse α-DnaK (Stressgen). Followed by an anti-mouse HRP conjugated secondary antibody (Sigma Aldrich). Membranes were then incubated with ECL reagent and visualised on a BioRad ChemiDoc XRS+system.

### Bacterial competition assays

Bacteria strains were cultured in LB media and harvested during the exponential growth phase. Subsequently, they were concentrated to OD_600_=1.0, mixed in a 10 : 1 ratio (attacker: prey bacteria) and competed against each other on an LB agar plate at 37 °C for 5 h. For the inducible T6SS strains, bacteria were competed against each other on a LB agar plate supplemented with 2 % (v/v) l-arabinose and 500 µm IPTG and incubated at 37 °C for 5 h. After competition, prey CFUs were recovered by plating serial dilutions onto selective media for the prey bacteria. *E. coli* (BZB1011) and the Δ*btub* derivative were selected for using nalidixic acid. SS381 however is resistant to nalidixic acid and therefore competition assays with SS381 as the attacker strain required the use of *E. coli* transformed with a selective plasmid (pULTRA-GFP-Km) to allow kanamycin selection of the *E. coli* prey.

### Colicin diffusion assays

Exponential phase indicator bacteria were inoculated into soft LA (0.7 % w/v) and overlaid on top of an LA plate (1.5 % w/v). Bacterial strains that were tested for colicin activity were harvested by centrifugation at 17500 ***g*** for 5 mins followed by filtration of the supernatant through sterile 0.22 µm filters. Supernatants were spotted onto the indicator bacteria plates and incubated overnight at 37 °C with zones of clearances indicating the presence of a diffusible anti-bacterial compound, i.e. colicins.

### Quantitative colicin plate reader assays

Overnight cultures of indicator strains *E. coli* (WT and *ΔbtuB*) or SS381 (WT and *ΔtolA*) were diluted to OD600=0.75. Then 100 µl of supernatant from each *S. sonnei* strain or EHEC was added to 100 µl of either *E. coli* WT or *E. coli ΔbtuB* in a 96 well plate in triplicate. The plate was incubated at 37 °C for 3 h with shaking in a plate reader to measure the OD_600_ of the *E. coli* indicator strains in the presence of the colicins.

### Preparation of culture supernatants for proteomics analysis

*S. sonnei* CIP106347 were diluted from overnight cultures and grown in LB until OD_600_=1, in triplicate. Cells were then incubated with or without mitomycin C (final concentration of 0.5 µg ml^−1^) at 37 °C for 2 h to induce colicin production. Bacteria were harvested by centrifugation at 4200 ***g*** for 10 min at 4 °C followed by filtration of the supernatant through sterile 0.22 µm filters and precipitated with trichloroacetic acid. Acetone was removed and the pellets air dried.

Proteins precipitated from 30 ml of culture supernatant were separated on a 10 % SDS-PAGE gel and visualised by Coomassie staining. SDS-PAGE bands were excised from the gel, destained in cycles of 50 mM ammonium bicarbonate and 50 mM ammonium containing 50 % (v/v) acetonitrile, dehydrated with acetonitrile and then reduced with 10 mM DTT and alkylated with 55 mM iodoacetamide (45 min each at 56 °C or at RT in the dark, respectively). Samples were washed and dehydrated then 40 ng of modified sequencing-grade trypsin (Promega, Fitchburg, MA, USA) was added on each dehydrated gel piece for overnight digestion at 37 °C. Resulting peptides were then extracted from the gel pieces in 60 % acetonitrile and 5 % formic acid and vacuum-dried before injection in mass spectrometry. The peptide mixtures were separated on a C-18 analytic column (75 µm ID ×25 cm nanoViper, 3 µm Acclaim PepMap) on an Easy-nanoLC-1000 system and analysed on a Q-Exactive Plus mass spectrometer (Thermo-Fisher Scientific, Bremen, Germany).

MS data were searched with Mascot (version 2.8.0.1, Matrix Science) against the databases of *S. sonnei*, contaminants (UniprotKB, version 2021_02, 22 219 and 111 sequences, respectively), as well as a homemade database of *E. coli* colicins’ sequences (48 sequences). Proteins were validated on Mascot rank equal to one, a Mascot score threshold set at 25, and 1 % FDR on both peptide spectrum matches (PSM) and protein sets (based on Mascot score) in ProlineStudio v2.1.2 (ProFI).

### Bioinformatics analysis

Easyfig (V2.2.2) [57] was used to generate the nucleotide synteny figure. Proksee was used to generate and annotate (integrated tools pLannotate v1.1.0 and Prokka v1.1.1) the CIP106347 spB plasmid map [58]. *S. sonnei* typing was performed using the open source genotyping tool (V20210201) based on the *Shigella* genotyping scheme [49].

### Statistical analysis

Statistical analysis was performed in Graphpad Prism (V.10.1.0). Statistical tests are described in figure legends. Data from competition assays were log transformed and statistical analysis performed on the transformed data. Significance values are annotated as ns=non significant, *P*≤0.05=*, *P*≤0.01=**, *P*≤0.001=***, *P*≤0.0001=****.
